# Decoding Membrane Lipids: Analytical Barriers and Technological Advances in Modern Lipidomics

**DOI:** 10.3390/ijms27031472

**Published:** 2026-02-02

**Authors:** Kyung-Hee Kim, Byong Chul Yoo

**Affiliations:** 1 Department of Applied Chemistry, School of Science and Technology, Kookmin University, Seoul 02707, Republic of Korea; kyungheekim@kookmin.ac.kr; 2 Antibody Research Institute, Kookmin University, Seoul 02707, Republic of Korea; 3 Diagnostic Research Team, InnoBation Bio R&D Center, Seoul 03929, Republic of Korea

**Keywords:** membrane lipids, lipidomics, phospholipid remodeling, acyl-chain composition, membrane asymmetry, lipid rafts, membrane curvature, phosphatidylserine, ceramides, extracellular vesicle

## Abstract

Biological membranes are dynamic, information-rich platforms whose structural and functional properties are dictated by lipid composition rather than acting as passive barriers. Recent advances in lipidomics have revealed that variations in lipid headgroups, acyl-chain length and saturation, sn-positional architecture, and oxidative modifications profoundly influence membrane mechanics, lateral organization, and protein–lipid interactions. These features collectively regulate fundamental cellular processes, including signaling, trafficking, curvature generation, and transbilayer asymmetry. In parallel, a wide range of pathological conditions—including cancer, metabolic disorders, neurodegeneration, and inflammatory diseases—are increasingly associated with coordinated lipid remodeling that reshapes membrane material properties and electrostatic landscapes. In this review, we integrate biophysical principles with lipidomics-based evidence to elucidate how lipid chemical diversity translates into membrane-level behavior. We discuss the roles of major membrane lipid classes, the functional consequences of acyl-chain and sn-positional remodeling, and the biological significance of lipid asymmetry and lateral heterogeneity. Particular attention is given to disease-associated lipid reprogramming and extracellular vesicle lipidomes as functional extensions of cellular membranes. Finally, we examine key analytical barriers in modern lipidomics and outline strategies required to connect lipid structural information with biological function. Together, this framework highlights membrane lipid architecture as a central determinant of cellular physiology and a promising axis for mechanistic insight and translational biomarker discovery.

## 1. Introduction

Functionally, lipid composition operates as a tunable “material parameter” that sets membrane mechanics (bending rigidity, compressibility, and line tension) and thereby constrains the conformational landscapes of embedded proteins [1,2,3,4]. In practice, lipid remodeling can rewire signaling and trafficking without any change in the proteome, simply by shifting the energetic cost of domain formation, curvature generation, or electrostatic recruitment [5,6,7].

Membrane lipids constitute a structurally diverse class of biomolecules that actively govern cellular architecture, membrane dynamics, and biological function rather than serving merely as passive structural components [8,9]. Variations in lipid headgroup chemistry, acyl-chain length and saturation, and sn-positional configuration collectively determine membrane thickness, curvature stress, surface charge, and lateral packing behavior [10,11].

A defining feature of biological membranes is their pronounced lipid asymmetry across bilayer leaflets, which is actively maintained by ATP-dependent flippases, floppases, and scramblases [12,13]. Disruption of this asymmetry has profound consequences for membrane biophysics and cellular signaling, influencing vesicle formation, membrane fusion, and cell–cell interactions [14,15].

Beyond compositional asymmetry, lateral heterogeneity within membrane leaflets gives rise to microdomains enriched in specific lipid classes, such as sphingolipids and cholesterol [16,17]. These microdomains modulate protein sorting, signal transduction, and membrane trafficking, underscoring the functional interdependence between lipid organization and cellular physiology [18].

Recent advances in mass spectrometry-based lipidomics have dramatically expanded our capacity to profile membrane lipid compositions at high structural resolution [19,20,21]. However, the inherent structural complexity of lipids—including extensive isomerism and overlapping mass spectra—poses significant analytical and interpretative challenges [22,23].

As a result, discrepancies often arise between measured lipid profiles and their inferred biological functions, particularly when subtle structural features such as double-bond position or sn-positional isomerism are overlooked [24,25]. Addressing these challenges requires an integrated framework that links lipid structural determinants to membrane biophysics, cellular function, and analytical constraints.

In this review, we synthesize current knowledge on membrane lipid structure, organization, and remodeling, with particular emphasis on how analytical limitations shape biological interpretation. By integrating structural lipid chemistry with membrane biology and lipidomics methodology, we aim to provide a coherent framework for understanding the functional consequences of membrane lipid diversity in health and disease.

Despite rapid advances in lipidomics, a unifying framework that explicitly links lipid structural determinants, membrane biophysics, and analytical limitations remains lacking. This Review addresses this gap by integrating structural lipid chemistry with membrane organization and disease-associated remodeling.

## 2. Structural Determinants of Membrane Lipids

**Figure 1:**
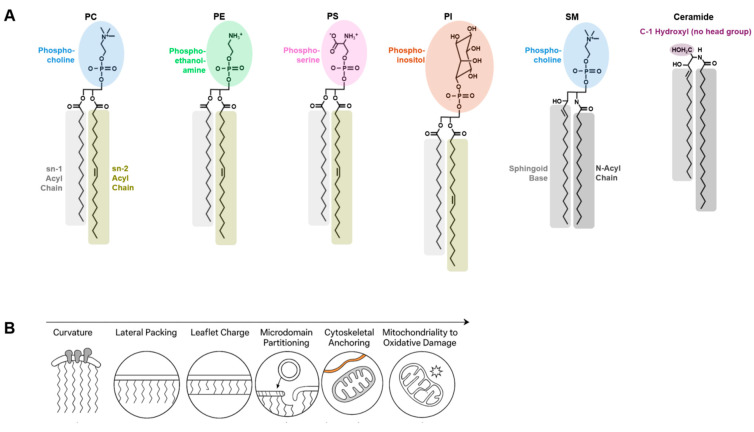
Structural and Functional Diversity of Membrane Lipids. (**A**) Overview of the major classes of membrane lipids and their key structural determinants, including headgroup chemistry, acyl-chain length and saturation, double-bond position, sn-positional configuration, and oxidative modifications. Representative lipid classes such as phosphatidylcholine (PC), phosphatidylethanolamine (PE), phosphatidylserine (PS), phosphatidylinositol (PI), sphingomyelin (SM), and ceramide are shown to illustrate structural diversity at the molecular level. (**B**) Functional consequences of lipid structural diversity at the membrane level. Variations in lipid geometry and chemical composition are shown to modulate membrane curvature, lateral packing, surface charge, and microdomain partitioning, thereby influencing protein recruitment, vesicle trafficking, organelle dynamics, and signaling. Phosphatidylserine is highlighted as a representative example of a lipid species that can exert disproportionate influence on membrane behavior under physiological and pathological conditions.

Figure 1 provides an integrated overview of how lipid headgroup chemistry, acyl-chain composition, sn-positional architecture, and oxidative modifications collectively shape membrane biophysical properties and emergent cellular functions.

For physicochemical context, the bending rigidity of biological membranes typically spans a range of approximately 10–40 kBT depending on lipid composition and cholesterol content, with cholesterol-rich plasma membranes being mechanically more rigid than most intracellular organelle membranes. Likewise, membrane thickness varies by several angstroms (Å) across organelles, reflecting differences in acyl-chain length and saturation.

### 2.1. Headgroup Chemistry and Interfacial Properties

Beyond net charge, headgroup-specific hydration shells and hydrogen-bonding networks influence interfacial viscosity and the residence time of peripheral proteins at the membrane surface [5,26]. For phosphoinositides, low-abundance species can exert outsized effects by creating high-avidity binding sites for polybasic domains, thereby coupling local lipid chemistry to actin remodeling and vesicle trafficking [6,27,28].

Lipid headgroups define the chemical identity of membrane lipids and exert a dominant influence on interfacial hydration, electrostatics, and hydrogen-bonding capacity at the membrane surface [8,9]. Zwitterionic phospholipids such as phosphatidylcholine (PC) and phosphatidylethanolamine (PE) differ markedly in headgroup size and hydrogen-bonding geometry, resulting in distinct effects on membrane packing and curvature stress [29,30].

Anionic lipids, including phosphatidylserine (PS) and phosphatidylinositol (PI), contribute negative surface charge to the membrane, thereby modulating electrostatic interactions with peripheral and integral membrane proteins [5,13]. These interactions are critical for the recruitment of signaling proteins and cytoskeletal components, particularly in membranes enriched in PS or polyphosphoinositides [26,27].

Headgroup chemistry also governs lipid–lipid interactions that influence lateral organization. For example, sphingomyelin (SM) headgroups form extensive hydrogen-bonding networks that promote tight packing and favor association with cholesterol, contributing to the formation of ordered membrane domains [10,16].

The major membrane lipid classes discussed in this section, together with their core structural features and disease relevance, are summarised in Table 1.

### 2.2. Acyl-Chain Length, Saturation, and sn-Positional Effects

From a biophysical perspective, hydrophobic mismatch between transmembrane helices and bilayer thickness can bias protein oligomerization and sorting, providing a mechanistic link between acyl-chain remodeling and receptor clustering [1,3]. Importantly, the functional impact of unsaturation depends not only on the number of double bonds but also on their position and geometry—features that frequently remain unresolved in standard lipidomics pipelines [33].

Beyond headgroups, the physicochemical properties of lipid acyl chains critically shape membrane structure. Acyl-chain length directly determines bilayer thickness, which in turn influences the conformational matching between membranes and transmembrane proteins [1,3]. Lipids with longer and more saturated acyl chains promote tighter packing and reduced membrane fluidity, whereas shorter or unsaturated chains increase bilayer flexibility [34].

The degree and geometry of unsaturation introduce kinks into acyl chains, reducing van der Waals interactions and increasing lateral diffusion within the membrane [2,35]. Polyunsaturated fatty acids, in particular, impart pronounced disorder and have been implicated in modulating membrane protein activity and signaling pathways [36].

Importantly, the asymmetric distribution of acyl chains between the sn-1 and sn-2 positions of glycerophospholipids adds an additional layer of structural complexity. Saturated acyl chains are preferentially found at the sn-1 position, whereas unsaturated chains are enriched at the sn-2 position, creating intrinsic molecular asymmetry that affects lipid packing and curvature propensity [11]. This sn-positional heterogeneity is often obscured in bulk lipidomic measurements but has significant implications for membrane mechanics and lipid–protein interactions [24,25].

As summarised in Table 2, the asymmetric distribution of saturated and unsaturated acyl chains between the sn-1 and sn-2 positions introduces intrinsic molecular asymmetry that directly impacts membrane fluidity, curvature stress, and vesicle dynamics.

### 2.3. Lipid Oxidation and Remodeling as Structural Modulators

Oxidative lipid modifications also complicate biological interpretation because oxidized species can act as signaling mediators while simultaneously perturbing bilayer organization, creating mixed “cause–effect” signatures in disease lipidomes [39,40]. Accordingly, rigorous analytical discrimination of oxidized positional isomers is essential to avoid conflating oxidative damage with adaptive remodeling [25,41].

Lipid structures are dynamically remodeled through enzymatic reactions and oxidative processes that alter both headgroup composition and acyl-chain characteristics. Phospholipase-mediated remodeling pathways, such as the Lands’ cycle, enable rapid turnover of acyl chains and facilitate adaptive responses to metabolic and environmental cues [37,38].

Oxidative modification of unsaturated lipids introduces polar functional groups into acyl chains, dramatically altering membrane permeability, curvature stress, and protein interactions [42,43]. Oxidized phospholipids accumulate under conditions of oxidative stress and have been implicated in inflammation, apoptosis, and disease-associated membrane dysfunction [39,40].

Together, headgroup chemistry, acyl-chain composition, sn-positional asymmetry, and post-synthetic modifications constitute a multidimensional structural space that governs membrane behavior. Disentangling the contributions of these determinants remains a central challenge for lipidomics and membrane biology, particularly given current analytical limitations in resolving isomeric and oxidized lipid species [21,23].

## 3. Membrane Organization and Lipid Asymmetry

### 3.1. Transbilayer Lipid Asymmetry and Its Maintenance

Mechanistically, ATP-dependent lipid translocases not only preserve asymmetry but also influence local membrane curvature by controlling the leaflet area difference, thereby intersecting with vesicle budding and endocytosis [12,13]. In pathological contexts, sustained PS exposure can reprogram cell–cell communication and clearance pathways, blurring the boundary between transient apoptotic signaling and chronic stress-associated membrane states [15,44].

Biological membranes exhibit a pronounced asymmetric distribution of lipid species between the inner and outer leaflets, a feature that is actively maintained and tightly regulated [9,13]. In the plasma membrane, aminophospholipids such as PS and PE are preferentially enriched in the cytosolic leaflet, whereas PC and SM are predominantly localized to the exoplasmic leaflet [12].

This transbilayer asymmetry is established and preserved by the coordinated activity of ATP-dependent flippases and floppases, as well as Ca^2+^-activated scramblases [14,45]. Disruption of these regulatory systems leads to aberrant lipid redistribution, which can profoundly alter membrane electrostatics, curvature, and protein interactions [15].

Loss of lipid asymmetry is not merely a structural perturbation but also serves as a biologically meaningful signal. Externalization of PS, for example, functions as a recognition cue during apoptosis and cell clearance, linking membrane organization directly to intercellular communication and immune regulation [44,46].

In addition to differences between the plasma membrane and extracellular vesicles, lipid composition varies markedly across subcellular organelles, reflecting organelle-specific functional and biophysical requirements. The plasma membrane is enriched in cholesterol, sphingomyelin, and relatively saturated phospholipids, conferring high lateral order, increased bilayer thickness, and mechanical robustness required for barrier function, signal transduction, and interaction with the extracellular environment [9,16,17,18]. In contrast, membranes of intracellular organelles such as the endoplasmic reticulum (ER) and Golgi apparatus are comparatively depleted in cholesterol and sphingolipids and enriched in unsaturated glycerophospholipids, resulting in thinner and more fluid bilayers that facilitate membrane deformation, vesicle budding, and high rates of lipid and protein trafficking [9,11,19].

Mitochondrial membranes exhibit an even more specialized lipid composition, characterized by enrichment of cardiolipin and phosphatidylethanolamine, which support high membrane curvature, tight packing of respiratory chain complexes, and efficient oxidative phosphorylation [11,20]. Similarly, endosomal and lysosomal membranes display distinct lipid profiles enriched in anionic lipids and sphingolipid metabolites that contribute to membrane stability under acidic conditions and regulate fusion, fission, and trafficking processes [14,16]. Importantly, these compositional differences are not passive consequences of lipid biosynthesis but are actively established and maintained through organelle-specific lipid synthesis, transport, and remodeling pathways [9,11]. Collectively, organelle-selective lipid enrichment links membrane composition to curvature stress, surface charge, protein recruitment, and enzymatic activity, underscoring lipid composition as a central determinant of organelle identity and function.

### 3.2. Lateral Heterogeneity and Membrane Microdomains

Domain formation should be viewed as a dynamic, nanoscale phenomenon in which transient assemblies emerge and dissolve on timescales relevant to signaling, rather than as stable “rafts” with fixed composition [7,17]. Lipid-driven partitioning can therefore amplify weak protein–protein interactions by locally concentrating receptors and adaptors, providing a plausible route from compositional heterogeneity to switch-like signaling outputs [16,47].

In addition to transbilayer asymmetry, membranes are laterally heterogeneous, exhibiting nanoscale organization into lipid microdomains with distinct compositional and physical properties [16]. These domains are typically enriched in sphingolipids and cholesterol, which together promote the formation of more ordered and tightly packed membrane regions [9].

Experimental and computational studies have demonstrated that such microdomains influence protein partitioning, trafficking, and signal transduction by providing specialized lipid environments [17,18]. Importantly, the stability and size of these domains are highly sensitive to lipid acyl-chain saturation and headgroup chemistry, underscoring the interconnectedness of lipid structure and membrane organization [48].

Although the raft concept has been refined over time, it remains clear that lateral lipid heterogeneity represents a fundamental organizing principle of membranes rather than an experimental artifact [7,47].

### 3.3. Curvature, Charge, and Functional Consequences

Curvature generation is often cooperative: cone-shaped lipids lower the energetic barrier for bending, while curvature-sensing proteins stabilize highly curved intermediates—together enabling efficient fission/fusion during trafficking [30,49]. This cooperation implies that relatively small shifts in PE content or sn-2 unsaturation can translate into large changes in vesicle production and membrane turnover [11,24].

Membrane organization is further shaped by curvature stress and surface charge, both of which are directly modulated by lipid composition. Cone-shaped lipids such as PE and lipids with intrinsic negative spontaneous curvature, including phosphatidic acid, favor membrane bending and facilitate vesicle budding and fusion [30,50,51]. In contrast, cylindrical lipids such as PC stabilize planar bilayers and resist curvature deformation [29].

Surface charge, largely determined by anionic lipids including PS and phosphoinositides, plays a critical role in recruiting cytosolic proteins through electrostatic interactions [5,27]. Changes in lipid composition that alter membrane charge density can therefore reprogram protein localization and signaling pathways without changes in protein expression levels [6].

Collectively, transbilayer asymmetry, lateral heterogeneity, curvature, and electrostatics define a dynamic organizational framework that enables membranes to integrate structural diversity with functional specificity. Capturing this complexity remains a central challenge for both experimental membrane biology and lipidomics-based analyses [7,17].

## 4. Lipid Remodeling in Disease and Extracellular Vesicles (EVs)

### 4.1. Metabolic Rewiring and Disease-Associated Lipid Remodeling

Systems-level lipidomics across large cohorts has underscored that disease signatures frequently reflect coordinated pathway shifts (e.g., de novo lipogenesis, elongation/desaturation, and phospholipid remodeling) rather than single-marker changes, reinforcing the need for pathway-aware interpretation [32,51]. These coordinated shifts can alter not only membrane material properties but also the availability of bioactive lipid mediators that feed back on inflammation and stress responses [36,49].

Cellular lipid composition is dynamically remodeled in response to metabolic and environmental cues, and dysregulation of these processes is increasingly recognized as a hallmark of disease [48,49]. Alterations in phospholipid class abundance, acyl-chain saturation, and remodeling enzyme activity have been reported across a broad spectrum of metabolic disorders, including obesity, diabetes, and non-alcoholic fatty liver disease [49,52].

In cancer, metabolic rewiring drives profound changes in lipid synthesis and remodeling pathways, leading to characteristic shifts in membrane lipid composition [53,54]. These changes support rapid cell proliferation by modulating membrane fluidity, signaling platform availability, and vesicular trafficking [55].

Importantly, disease-associated lipid remodeling often involves subtle structural changes—such as altered sn-positional distributions or selective enrichment of unsaturated acyl chains—that are not readily captured by bulk lipid measurements [23,24]. As a result, functional consequences of lipid remodeling may be underestimated when analytical resolution is limited.

Representative examples of disease-associated lipid structural remodeling and their functional consequences are summarised in Table 3.

### 4.2. Lipid Dysregulation and Membrane Function in Cancer

An emerging conceptual model is that tumor cells exploit lipid remodeling to balance competing constraints—maintaining sufficient fluidity for trafficking and division while increasing rigidity in selected regions to resist oxidative damage and immune attack [54]. Such spatially heterogeneous remodeling aligns with the idea that microdomain organization and electrostatic landscapes are actively tuned to favor oncogenic signaling complexes [16].

Cancer cells exhibit coordinated upregulation of de novo lipogenesis and lipid uptake pathways, resulting in membranes that are compositionally distinct from those of non-transformed cells [55,57]. Enhanced incorporation of saturated and monounsaturated fatty acids into membrane phospholipids has been linked to increased membrane rigidity and resistance to oxidative stress [54].

At the same time, selective enrichment of specific lipid classes, including phosphatidylserine and phosphoinositides, reshapes membrane electrostatics and signaling capacity in tumor cells [27]. These compositional changes influence not only intrinsic signaling pathways but also interactions with the tumor microenvironment and immune system [14,31,58].

Such observations underscore the need to interpret cancer-associated lipid alterations within a structural and functional framework, rather than as isolated changes in lipid abundance.

### 4.3. EV Lipidomes as Functional Extensions of the Cell Membrane

Because EV preparations are often contaminated by lipoproteins and other nanoparticles, lipid composition can be strongly skewed by isolation method, making adherence to reporting and methodological standards essential for cross-study comparability [59,60,61]. Conceptually, EV lipidomes can be interpreted as ‘exported membrane states’ that capture both biogenesis pathways and parent-cell stress, offering a route to noninvasive readouts of membrane remodeling in disease [62,63].

EVs, including exosomes and microvesicles, represent membrane-bound carriers of bioactive molecules that mediate intercellular communication [60,62]. EV membranes are not passive reflections of the parent cell membrane but are selectively enriched in specific lipid species, including sphingolipids, cholesterol, and anionic phospholipids, conferring distinct biophysical properties and biological functions [61,62].

Lipidomic analyses have revealed that EV lipid composition is shaped by both biogenetic pathways and disease state, with cancer-derived EVs often displaying distinct lipid signatures that influence vesicle stability, uptake, and biological activity [61,64]. These lipid features contribute to EV-mediated modulation of immune responses, angiogenesis, and metastatic niche formation [63,65].

However, accurate characterization of EV lipidomes remains challenging due to their small size, compositional heterogeneity, and susceptibility to analytical bias [23,60]. These limitations complicate the interpretation of EV lipidomics data and highlight the need for standardized analytical workflows.

## 5. Analytical Barriers and Interpretative Challenges in Lipidomics

The major analytical barriers that complicate structural and quantitative interpretation of lipidomic data are summarised in Table 4.

**Figure 2:**
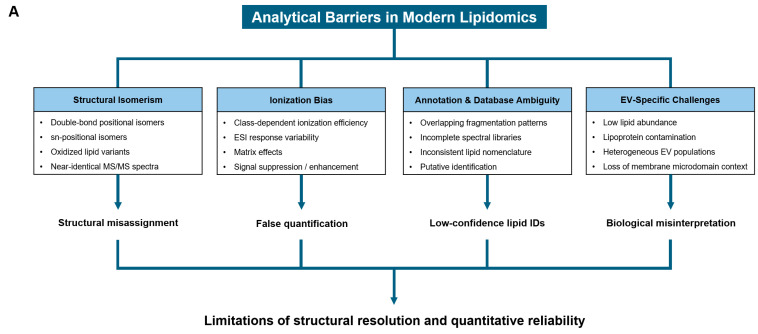

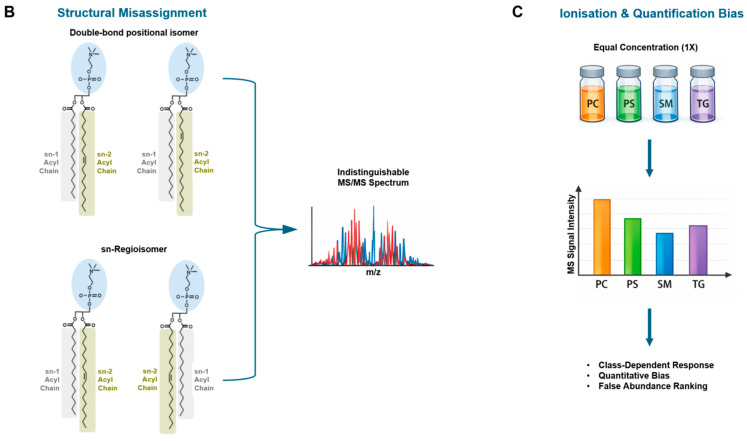
Analytical Barriers in Structural and Quantitative Lipidomics. This schematic summarises the major analytical challenges that limit structural resolution and quantitative reliability in modern lipidomics, with direct implications for biological interpretation and clinical translation. (**A**) Double-bond positional isomerism and sn-regioisomerism. Lipids sharing identical molecular formulas but differing in double-bond position or sn-1/sn-2 acyl-chain configuration generate nearly indistinguishable MS/MS fragmentation patterns. This isomeric complexity hampers confident structural annotation, particularly for glycerophospholipids such as PC, PE, and PS. (**B**) Structural misassignment due to insufficient resolution. Overlapping fragment ions among isomeric species lead to ambiguous lipid identification and low-confidence lipid assignments. Consequently, structurally distinct lipids may be incorrectly grouped or misannotated, propagating errors into downstream biological interpretation. (**C**) Ionisation-dependent quantitative bias. Even at equal molar concentrations (1×), lipid classes exhibit markedly different electrospray ionisation efficiencies, resulting in class-dependent MS signal intensities. This ionisation variability introduces systematic quantitative bias, distorts relative abundance ranking, and leads to false quantification across lipid classes. Together, these analytical limitations illustrate how inadequate structural resolution and ionisation bias can propagate from lipid misidentification to erroneous quantification and ultimately to biological misinterpretation, underscoring the need for advanced structural lipidomics approaches.

Figure 2 schematically illustrates how structural isomerism, ionisation bias, annotation ambiguity, and EV-specific challenges collectively limit the robustness and reproducibility of lipidomics-based measurements.

### 5.1. Structural Isomerism and Hidden Molecular Diversity

Practically, collapsing multiple isomers into a single reported feature can inflate apparent associations or mask mechanistically meaningful changes, especially when different isomers have opposing biophysical effects [24,25]. Therefore, study design should explicitly match biological questions to the structural resolution required, rather than treating ‘lipid class + total carbons:double bonds’ as universally sufficient [23,67].

A fundamental challenge in lipidomics arises from the extraordinary structural diversity of lipid molecules, including regioisomerism, stereoisomerism, and variations in double-bond position and geometry [22,23]. Lipids that are isobaric or even isomeric often share identical nominal masses and similar fragmentation patterns, rendering them difficult to distinguish using conventional mass spectrometry workflows [24,25].

This limitation is particularly consequential for glycerophospholipids, where differences in sn-positional acyl-chain distribution can profoundly affect membrane properties yet remain unresolved in many lipidomic datasets [11,50]. As a result, biologically meaningful structural information is frequently collapsed into aggregate lipid species, obscuring mechanistic interpretation. In routine LC–MS workflows, most glycerophospholipid isomers remain unresolved at the level of double-bond position or sn-regioisomerism, whereas advanced approaches such as ion mobility or ozone-induced dissociation can partially resolve these features under optimized conditions.

Recent methodological advances, including ion mobility spectrometry and ozone-induced dissociation, have begun to address these challenges by enabling partial resolution of lipid isomers [33]. Nevertheless, these approaches are not yet widely implemented, and their integration into routine lipidomics remains limited.

### 5.2. Ionization Bias and Quantitative Uncertainty

Quantitative robustness improves when class-matched internal standards are paired with transparent reporting of calibration strategies and limits of quantification, yet such practices remain inconsistent across the literature [19,23]. For clinical translation, harmonization efforts and interlaboratory QC frameworks are likely as important as incremental gains in instrument sensitivity [41].

Ionization efficiency varies widely among lipid classes and molecular species, introducing systematic bias into mass spectrometry-based quantification [21,66]. Differences in headgroup polarity, acyl-chain composition, and adduct formation can lead to preferential detection of certain lipid species, while others are underrepresented or missed entirely [19].

These biases complicate cross-study comparisons and can distort inferred biological trends, particularly when relative abundances are interpreted without appropriate internal standards [23]. Even with isotope-labeled standards, comprehensive coverage across the lipidome is rarely achieved, necessitating cautious interpretation of quantitative results.

Ion suppression effects further exacerbate these issues in complex biological matrices, where co-eluting species compete for ionization and reduce analytical sensitivity [40,68].

### 5.3. Annotation, Databases, and Biological Interpretation

Annotation quality is increasingly shaped by community standards and machine-readable vocabularies that reduce ambiguity in lipid naming and structural claims [23,69]. Ultimately, biological interpretation should integrate confidence levels for identification with orthogonal evidence (e.g., retention time, ion mobility, or targeted validation) to prevent ‘database-driven biology’ from outrunning analytical certainty [67,68].

Beyond analytical measurement, lipid identification and annotation present additional bottlenecks in lipidomics workflows. Automated database matching often assigns lipid identities based on limited spectral evidence, leading to overannotation or misannotation of lipid species [69,70]. Such errors can propagate through downstream analyses and undermine biological conclusions.

Efforts to standardize lipid nomenclature and reporting practices, exemplified by the Lipidomics Standards Initiative, represent important steps toward improving reproducibility and transparency [23,67]. However, widespread adoption of these standards remains incomplete, and inconsistencies persist across platforms and studies.

Critically, analytical uncertainty must be considered alongside biological context. Without integrating structural resolution, quantitative confidence, and mechanistic understanding, lipidomics data risk being overinterpreted or misapplied [68,69]. Addressing these challenges will be essential for translating lipidomic insights into robust biological and clinical conclusions.

## 6. Conclusions and Outlook

A practical implication for future studies is to couple hypothesis-driven perturbations (e.g., remodeling enzyme modulation or controlled oxidative stress) with structurally resolved lipidomics, enabling causal links between defined structural features and membrane-level phenotypes [38,40]. In parallel, EV lipidomics will benefit from standardized isolation and reporting pipelines that preserve interpretability across cohorts and platforms [23,60].

**Figure 3:**
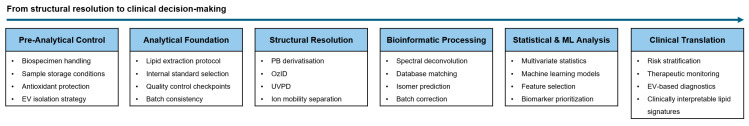
Integrated Workflow for Translational, Clinically Oriented Lipidomics. This figure presents an integrated workflow linking structurally resolved lipidomics to translational and clinical applications. Pre-analytical variables—including biospecimen handling, EV isolation strategies, antioxidant protection, and storage conditions—are shown to influence lipid integrity and data quality. The analytical tier highlights extraction strategies, internal standard selection, and quality-control checkpoints, followed by advanced mass spectrometry approaches for isomer resolution, such as Paternò–Büchi derivatisation, ozone-induced dissociation, ultraviolet photodissociation, and ion-mobility separations. Downstream bioinformatic modules incorporate spectral deconvolution, database matching, isomer-aware annotation, batch correction, and pathway-level mapping, which feed into statistical and machine-learning frameworks for biomarker discovery. The final clinical tier illustrates applications in risk stratification, therapeutic monitoring, and EV-based diagnostics, providing a conceptual bridge between high-resolution lipidomics and precision medicine.

Figure 3 outlines an integrated, clinically oriented lipidomics workflow that links pre-analytical control, structurally resolved mass spectrometry, and bioinformatic interpretation to translational and diagnostic applications.

Membrane lipids are not passive structural components but active determinants of cellular architecture, dynamics, and function. As highlighted throughout this Review, subtle variations in lipid headgroup chemistry, acyl-chain length and saturation, sn-positional configuration, and post-synthetic modification collectively define membrane biophysical properties, including thickness, curvature stress, surface charge, and lateral organization [8,9,11].

A central theme emerging from recent studies is that lipid structural diversity is tightly linked to biological specificity. Transbilayer asymmetry, lateral heterogeneity, and curvature-dependent lipid sorting provide mechanistic frameworks through which membranes coordinate signaling, trafficking, and intercellular communication [7,13,16]. Perturbation of these organizational principles, whether through metabolic rewiring or pathological stress, leads to disease-associated lipid remodeling that cannot be fully captured by bulk compositional analyses alone [32].

Advances in mass spectrometry-based lipidomics have substantially expanded our ability to profile membrane lipidomes, yet analytical barriers remain a major constraint on biological interpretation. Structural isomerism, ionization bias, and annotation uncertainty continue to obscure functionally relevant lipid features, particularly in complex systems such as cancer cells and EVs [23,24]. These limitations underscore the need to interpret lipidomics data within a rigorous structural and biophysical framework.

Recent advances in analytical methodologies have significantly extended the capabilities of lipidomics. Comprehensive reviews have highlighted the development of enhanced chromatographic separation techniques, higher mass spectrometric resolution, and refined data processing workflows that together enable more sensitive and accurate profiling of lipid species across diverse biological matrices [71]. Moreover, combining liquid chromatography with ion mobility mass spectrometry has yielded fast, broad-coverage lipidomic workflows capable of better resolving complex and isobaric lipid species, thereby addressing long-standing analytical challenges associated with lipid structural diversity [72]. These technological innovations continue to push the frontier of lipidomics toward higher dimensional analyses, improved isomer discrimination, and more comprehensive characterization of biological lipidomes.

Looking forward, progress in membrane lipid biology will depend on closer integration between analytical innovation and biological hypothesis testing. Improved structural resolution, standardized reporting practices, and quantitative confidence are essential for translating lipidomic measurements into mechanistic insight [21,69]. Ultimately, a determinant-centered view of membrane lipids—one that links molecular structure to membrane organization and function—will be critical for advancing our understanding of lipid-mediated processes in physiology and disease.

## Figures and Tables

**Table 1:** Major Membrane Lipid Classes and Core Biological Roles.

Lipid Class	Key Structural Feature	Primary Membrane Role	Disease Relevance	Key Reference
PC	Zwitterionic, cylindrical	Outer leaflet stability	ER stress, cancer remodeling	[9]
PE	Cone-shaped headgroup	Curvature generation, fusion	Mitochondrial dysfunction	[11]
PS	Anionic, inner leaflet-enriched	Signaling scaffold, electrostatics	Immune evasion in cancer	[14,31]
SM	Saturated acyl chains, H-bonding	Ordered domain (raft) formation	RTK clustering, viral entry	[16]
Ceramide	Long-chain sphingolipid backbone	Stress signaling, domain coalescence	Insulin resistance, apoptosis	[32]

PC, Phosphatidylcholine; PE, Phosphatidylethanolamine; PS, Phosphatidylserine; SM, Sphingomyelin; ER, Endoplasmic reticulum; RTK, Receptor tyrosine kinase.

**Table 2:** Acyl-Chain and sn-Positional Architecture of Membrane Lipids.

Feature	sn-1 Position	sn-2 Position	Biophysical Impact	Key Reference
Fatty acid type	Saturated fatty acid	Unsaturated/PUFA	Fluidity and curvature control	[11,37,38]
Enzymatic turnover	Low (PLA1)	High (PLA2)	Rapid signaling remodeling	[37,38]
Membrane effect	Rigid packing	Negative curvature stress	Vesicle formation, fusion	[29]

PUFA, Polyunsaturated fatty acid; PLA, Phospholipase A.

**Table 3:** Disease-Associated Lipid Structural Remodeling.

Structural Change	Molecular Consequence	Affected System	Clinical Implication	Key Reference
sn-2 PUFA depletion	↓ membrane flexibility	Cancer, brain	Progression, neurodegeneration	[36]
PS externalization	Increased surface negative charge	Tumor microenvironment	Immune evasion	[15,31]
Ceramide accumulation	Domain clustering, rigidity	Metabolic tissues	Insulin resistance, apoptosis	[32,56]
OxPL increase	Pro-inflammatory signaling	Vasculature, central nervous system	Chronic inflammation	[41]

PUFA, Polyunsaturated fatty acid; PS, Phosphatidylserine; OxPL, Oxidized phospholipid.

**Table 4:** Analytical Barriers in Modern Lipidomics.

Barrier Category	Underlying Cause	Impact on Data Quality	Most Affected Lipid Types	Key Reference
Structural isomerism	Double bond position, sn-heterogeneity, oxidation	Misannotation, false positives	OxPLs, plasmalogens, ceramides	[19,25]
Ionization bias	Class-specific ESI response	Quantification errors	PS, PI, TG, CE, EV lipids	[21,66]
Database limitations	Incomplete spectral libraries	Over/under-annotation	Ether lipids, bacterial lipids	[23]
EV lipidomics challenges	Low abundance; contamination	Biological misinterpretation	EV PS, ceramides, raft lipids	[60,61]

ESI, Electrospray ionization; OxPL, Oxidized phospholipid; PS, Phosphatidylserine; PI, Phosphatidylinositol; TG, Triacylglycerol; CE, Cholesteryl ester; EV, Extracellular vesicle.

## Data Availability

No new data were created or analyzed in this study. Data sharing is not applicable to this article.

## Abbreviations

CE	Cholesteryl Ester
ER	Endoplasmic Reticulum
ESI	Electrospray Ionization
EV	Extracellular Vesicle
MS	Mass Spectrometry
OxPL	Oxidized Phospholipid
PB	Paternò–Büchi (derivatization)
PC	Phosphatidylcholine
PE	Phosphatidylethanolamine
PI	Phosphatidylinositol
PLA	Phospholipase A
PS	Phosphatidylserine
PUFA	Polyunsaturated Fatty Acid
RTK	Receptor Tyrosine Kinase
SM	Sphingomyelin
TG	Triacylglycerol
UVPD	Ultraviolet Photodissociation
OzID	Ozone-Induced Dissociation
